# Genome-wide analysis of the citrus B3 superfamily and their association with somatic embryogenesis

**DOI:** 10.1186/s12864-020-6715-9

**Published:** 2020-04-16

**Authors:** Zheng Liu, Xiao-Xia Ge, Xiao-Meng Wu, Qiang Xu, Ross G. Atkinson, Wen-Wu Guo

**Affiliations:** 1 Fruit and Tea Research Institute, Hubei Academy of Agricultural Sciences, Wuhan, 430064 China; 2 Key Laboratory of Horticultural Plant Biology (Ministry of Education), Huazhong Agricultural University, Wuhan, 430070 China; 3 Center of Applied Biotechnology, Wuhan University of Bioengineering, Wuhan, 430415 China; 4 The New Zealand Institute for Plant & Food Research Limited (PFR), Private Bag 92169, Auckland, 1142 New Zealand

**Keywords:** Citrus, B3 superfamily, Phylogenetic analysis, Somatic embryogenesis, Callus initiation, Expression profile

## Abstract

**Background:**

In citrus, genetic improvement via biotechnology is hindered by the obstacle of in vitro regeneration via somatic embryogenesis (SE). Although a few B3 transcription factors are reported to regulate embryogenesis, little is known about the B3 superfamily in citrus, and which members might be involved in SE.

**Results:**

Genome-wide sequence analysis identified 72 (*CsB3*) and 69 (*CgB3*) putative B3 superfamily members in the genomes of sweet orange (*Citrus sinensis*, polyembryonic) and pummelo (*C. grandis*, monoembryonic), respectively. Genome duplication analysis indicated that segmental and tandem duplication events contributed to the expansion of the B3 superfamily in citrus, and that the B3 superfamily evolved under the effect of purifying selection. Phylogenetic relationships were well supported by conserved gene structure and motifs outside the B3 domain, which allowed possible functions to be inferred by comparison with homologous genes from *Arabidopsis*. Expression analysis identified 23 B3 superfamily members that were expressed during SE in citrus and 17 that may play functional roles at late SE stages. Eight B3 genes were identified that were specific to the genome of polyembryonic sweet orange compared to monoembryonic pummelo. Of these eight B3 genes, *CsARF19* was found to be specifically expressed at higher levels in embryogenic callus (EC), implying its possible involvement in EC initiation.

**Conclusions:**

This study provides a genome-wide analysis of the citrus B3 superfamily, including its genome organization, evolutionary features and expression profiles, and identifies specific family members that may be associated with SE.

## Background

B3 transcription factors (TFs), which contain at least one B3 DNA-binding domain, constitute one of the plant-specific superfamilies [1, 2]. The B3 domain was initially named according to its position in the third basic region of *VIVIPAROUS1* (*VP1*) from maize [3]. The conserved B3 domain comprises approximately 110 amino acid residues for DNA recognition, consisting of seven β-barrels and two short α-helices [1, 2]. According to domain structure and phylogenetic analysis, the B3 superfamily is divided into four major families, namely the LAV (LEAFY COTYLEDON2-ABSCISIC ACID INSENSITIVE3-VAL), RAV (RELATED TO ABI3/VP1), ARF (AUXIN RESPONSE FACTOR) and REM (REPRODUCTIVE MERISTEM) families [1]. The B3 superfamily has been characterized in a number of model plants and crops, including *Arabidopsis*, rice, poplar, *Brassica rapa*, castor bean, cocoa, soybean, maize, tobacco, grapevine, moss and algae [1, 4–7], but not yet in citrus.

It is reported that B3 TFs from distinct families regulate and control different aspects of plant growth and development. LAV family members, including *LEC2* (*LEAFY COTYLEDON2*), *FUS3* (*FUSCA3*), *ABI3* (*ABSCISIC ACID INSENSITIVE3*), *VAL1* (*VP1/ABI3-LIKE 1*), *VAL2* and *VAL3*, which each possess a single B3 domain, regulate callus induction, embryo development and phase transition [8–16]. For instance, overexpression of *AtLEC2* in transgenic plants induced the formation of callus and somatic embryos [9]. The LAV family generally consists of two subgroups: the LEC2-ABI3 subgroup (*LEC2*, *ABI3* and *FUS3*) recognizes the Sph/RY motif (CATGCA) in the promoters of seed-specific genes [4, 5, 17], whereas genes in the other subgroup VAL (*VAL1*, *VAL2* and *VAL3*) which also encode a CW-type zine finger, are expressed in many organs throughout plant development and have central roles in mediating repression of the *LEC1/LEC2-ABI3* subgroup network during seed germination [1, 6, 11, 18]. RAV family proteins contain a C-terminal B3 domain that recognizes the consensus sequence CACCTG [19]. Some members of the RAV family also possess an N-terminal AP2/ERF domain that recognizes the consensus sequence CAACA. RAV family members control flowering, organ growth, and have also been shown to be involved in leaf senescence, hormone signaling and responses to various stresses [19–26]. The ARF family proteins have an N-terminal B3 domain that recognizes the auxin response element TGTCTC in the promoter of genes responsive to auxin, followed by a highly divergent middle region that determines whether the ARFs act as an activator or repressor [27, 28]. Some ARF proteins contain a conserved carboxyl-terminal interaction domain (Aux/IAA), which is responsible for the dimerization [1]. ARF genes have been widely implicated in auxin-mediated responses during various developmental processes from embryogenesis to flowering, and fruit development [29–35]. REM family members contain at least one copy of the B3 domain, and sometimes up to seven repeats. However, it is not clear whether the B3 domain of the REM protein binds to a specific recognition sequence [36]. The function of REM genes are generally not well understood. Some genes including *REM1*, *VRN1* and *VOD* have been shown to be involved in floral meristem formation, vernalization and female gametophyte development [37–39].

Citrus is one of the most important fruit crops in the world. However, conventional breeding of citrus is hindered by characteristics such as nucellar polyembryony, long juvenility and male/female sterility [40]. Genetic improvement via biotechnology could be an effective approach, but it is hindered by the barrier of plant regeneration through somatic embryogenesis (SE). Embryogenic callus (EC) can only be induced from the aborted seeds of polyembryonic (apomictic) citrus genotypes, but not from monoembryonic (sexual) genotypes. In addition, the embryonic potential of EC gradually decreases during callus subculture. To understand the mechanisms of SE and overcome the obstacle of citrus SE, we have conducted a series of studies to identify genes, proteins and miRNAs involved in citrus SE [41–43]. We found that the B3 domain regulatory network genes *CsFUS3*, *CsABI3* and another B3 gene (CS_P006_E_03) exhibited increased expression during citrus SE induction and formation [41], whereas *CsFUS3* was shown to promote citrus SE by regulating SE-related TFs and hormone pathways, especially ABA and GA pathways [44]. In this study, we performed a genome-wide analysis of the B3 superfamily in polyembryonic sweet orange and monoembryonic pummelo to better understand the regulatory roles of the B3 superfamily genes in citrus SE This comprehensive study of the B3 superfamily should enhance our understanding of possible roles of B3 genes in citrus development, especially in SE.

## Results

### Identification and genomic distribution of B3 superfamily in citrus

A total of 72 (*CsB3*) and 69 (*CgB3*) B3 superfamily TFs were identified in the sweet orange (*Citrus sinensis*) and pummelo (*C. grandis*) genomes, respectively (Additional file 1). B3 superfamily members were classified into LAV, RAV, ARF and REM families, then systematically named according to their sequence similarity. In citrus, REM was found to be the biggest B3 family, with 52.8% (38 *CsREMs*) and 55.1% (38 *CgREMs*) of the total B3 genes identified in sweet orange and pummelo, respectively (Additional file 1). ARFs constituted the second largest family with 26.4% (19 *CsARFs*) and 24.6% (17 *CgARFs*) of the B3 genes in sweet orange and pummelo. The LAV and RAV families were much smaller, with 11.1% (8 *CsLAVs*) and 9.7% (7 *CsRAVs*) of B3 genes identified in sweet orange, and 11.6% (8 *CgLAVs*) and 8.7% (6 *CgRAVs*) of B3 genes identified in pummelo.

**Fig. 1:**
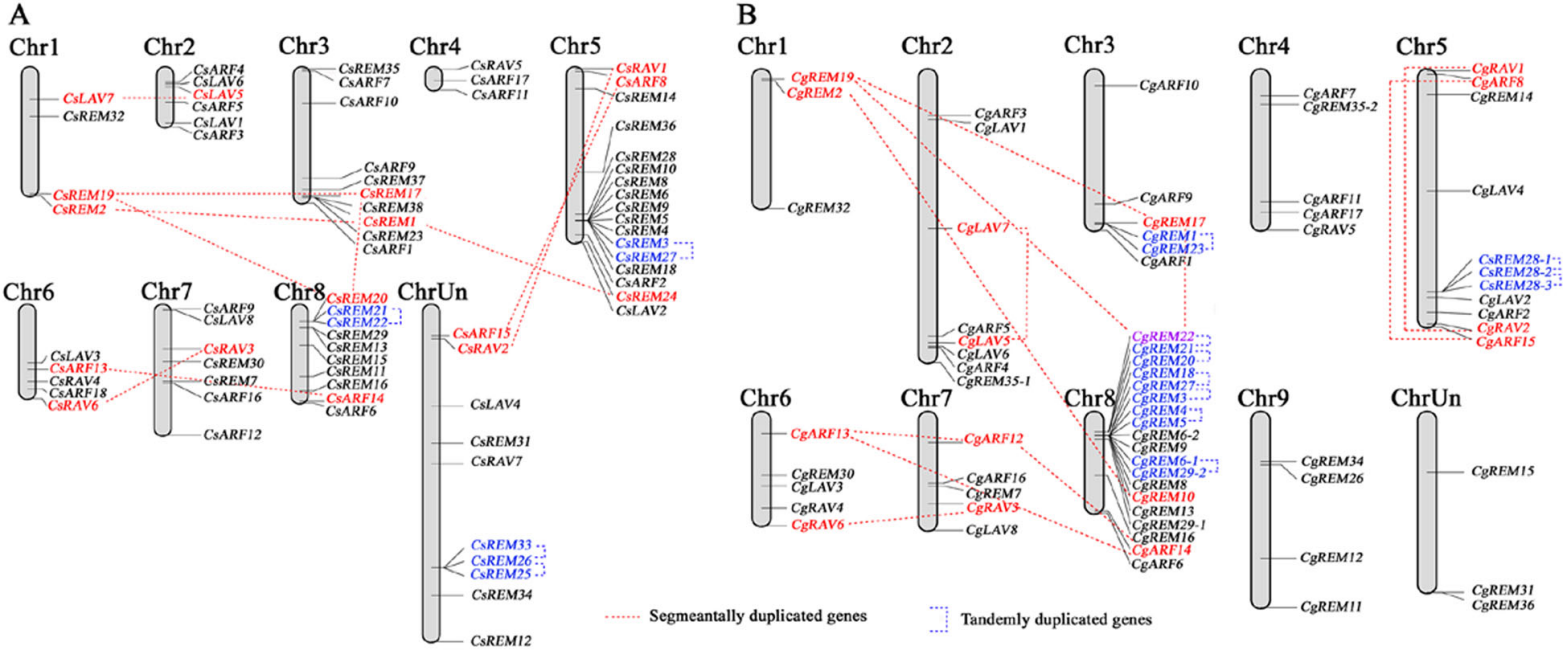
Chromosomal locations and regional duplication of citrus B3 genes. The chromosomal position of each B3 gene was mapped to the sweet orange (**a**) and pummelo (**b**) genomes. The chromosome number is indicated at the top of each chromosome. Segmentally duplicated gene pairs are linked by red dotted lines, whereas tandemly duplicated gene pairs are linked by blue dotted lines

*CsB3* TFs were distributed over eight of the nine sweet orange chromosomes. None of the *CsB3* genes was located on chromosome 9 (Fig. 1a). The *CsB3* gene density per chromosome was variable, with only three genes (4.2%) (namely *CsRAV5*, *CsARF11* and *CsARF17*) on chromosome 4, but up to 17 (23.6%) of the 72 members on chromosome 5. Relatively high densities of *CsB3* genes were observed at the chromosome ends, with the highest density at the bottom of chromosome 5. However, it should be noted, the chromosomal locations for 10 *CsB3* genes were not defined because of the incompleteness of sweet orange physical genome map. The distribution and density of *CgB3* TFs was also not uniform on the nine chromosomes of pummelo (Fig. 1b). Chromosome 8 contained the largest number of 19 (27.5%) *CgB3* genes, whereas on chromosome 1 there were only three (4.3%) *CgB3* genes.

Orthologous genes of the B3 superfamily between sweet orange and pummelo were not located consistently on the same citrus chromosomes. For example, *CsLAV7* was on chromosome 1 of sweet orange (Fig. 1a), whereas its orthologous gene *CgLAV7* was on chromosome 2 of pummelo (Fig. 1b). These different locations of B3 TFs on chromosomes between citrus species indicated that genetic recombination has occurred extensively in citrus varieties. Among all identified *CsB3* genes, a total of ten chromosomal segmental duplication events and four tandem duplication events were identified in the sweet orange genome, whereas in the pummelo genome the corresponding events were 11 and nine respectively (Fig. 1 and Additional file 2), indicating that segmental and tandem duplications may have contributed to the expansion of citrus B3 superfamily. Segmentally duplicated gene pairs (average Ka/Ks = 0.22, where Ka/Ks is the non-synonymous/synonymous substitution ratio) appeared to have undergone extensive intense purifying selection compared to tandemly duplicated gene pairs (average Ka/Ks = 0.52). The Ka/Ks ratios for the majority (82.4%) of the duplicated pairs were less than 0.5, suggesting that the citrus B3 superfamily has evolved under the effect of purifying selection. However, the other two tandemly duplicated gene pairs (*CgREM28–1/CgREM28–2* and *CgREM6–1/CgREM29–2*) seemed to be under neutral selection, as their Ka/Ks ratios were close to 1.0.

**Fig. 2:**
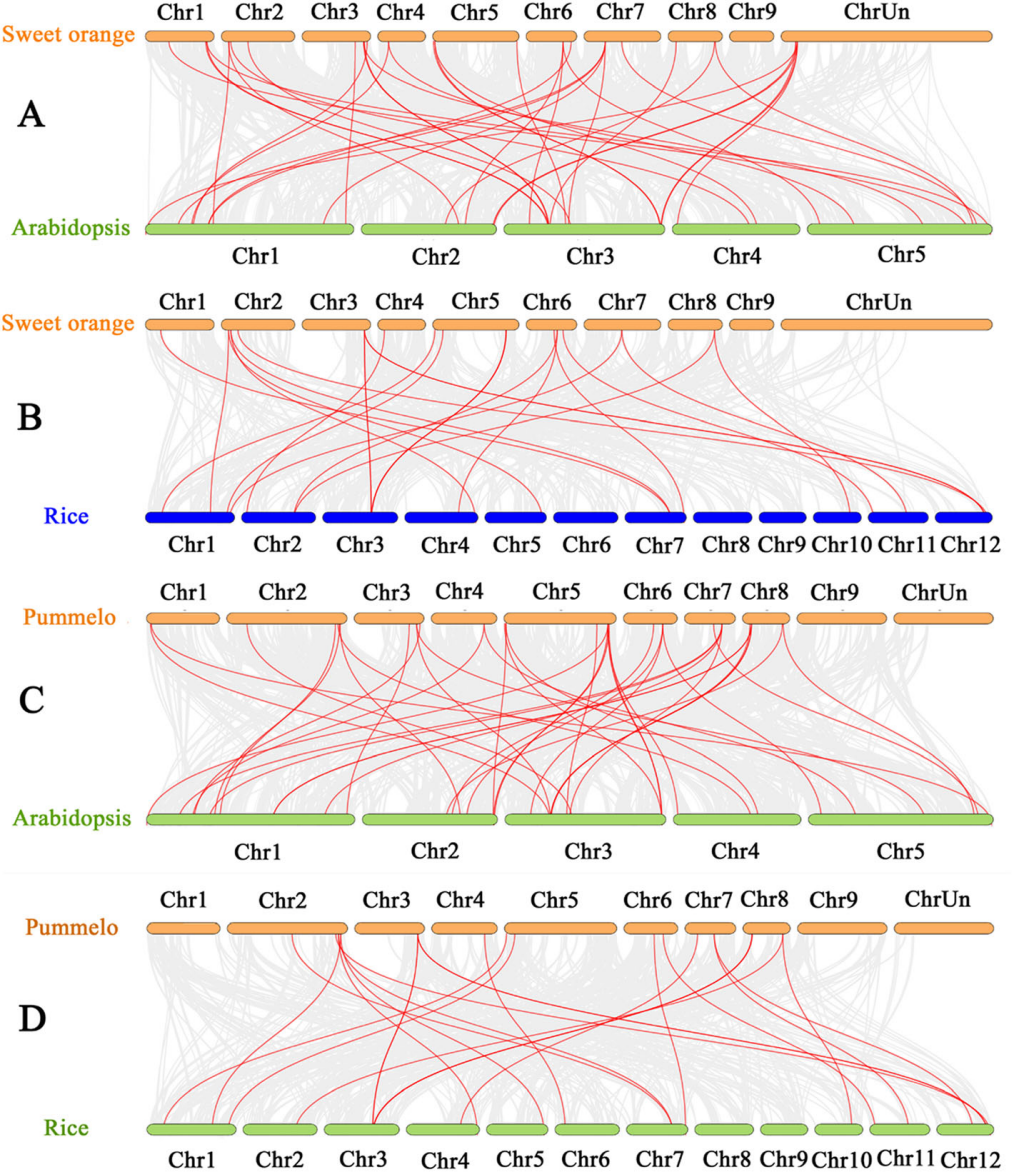
Gene duplication and synteny analysis of the B3 genes between sweet orange/pummelo and *Arabidopsis*/rice**.** Gray lines in the background indicate the collinear blocks within sweet orange/*Arabidopsis* genomes (**a**), sweet orange/rice genomes (**b**), pummelo/*Arabidopsis* genomes (**c**), and pummelo/rice genomes (**d**), respectively. The red lines highlight the syntenic B3 gene pairs

To further explore the relationship of B3 superfamily genes between citrus and other plant species, comparative syntenic analyses were conducted in a pairwise manner (Fig. 2), with 37 and 24 collinear B3 gene pairs identified in the sweet orange/*Arabidopsis* and sweet orange/rice pairs, respectively (Additional file 3). For pummelo/*Arabidopsis* and pummelo/rice comparisons the corresponding gene pair numbers were 39 and 24. The number of orthologous events of *CsB3/CgB3*-*AtB3* was higher than that of *CsB3/CgB3*-*OsB3*, indicating that the divergence between citrus and *Arabidopsis* occurred after the divergence of rice and the common ancestor of dicotyledons. It was noteworthy that some B3 collinear gene pairs of citrus/*Arabidopsis* were anchored to highly conserved syntenic blocks, in which the number of syntenic gene pairs was up to 246, whereas none of syntenic blocks of citrus/*Oryza sativa* pairs contained more than 20 genes (Additional file 3). The high level of syntenic conservation between the citrus and *Arabidopsis* indicated that B3 TFs in citrus might share similar structures and functions with orthologs in *Arabidopsis*.

### Characterization of B3 proteins in citrus

**Fig. 3:**
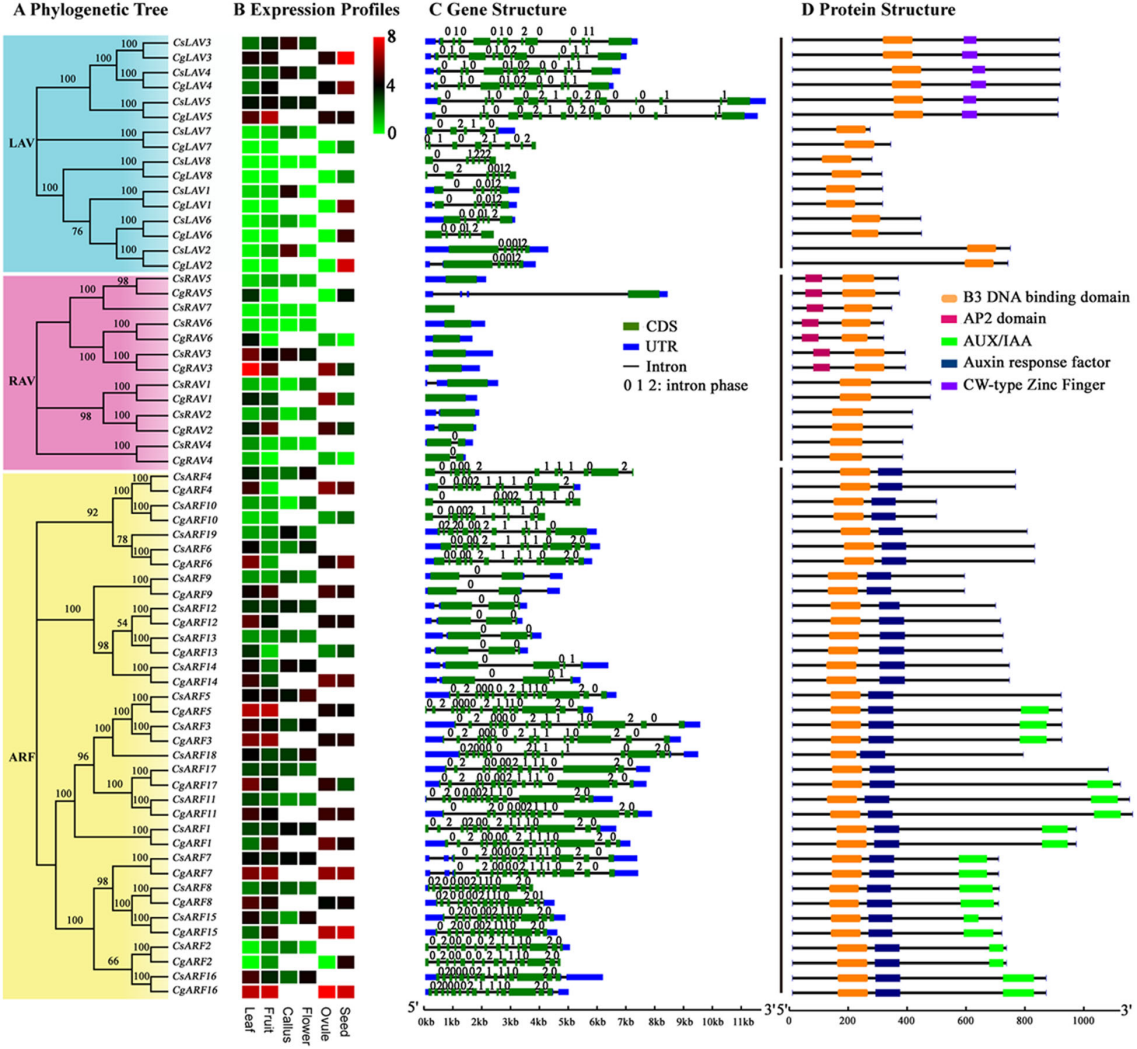
Phylogenetic relationships, expression profiles, gene structure and protein structure of citrus B3 genes from the LAV, RAV and ARF families. **a** Neighbor-joining trees constructed for B3 genes from the LAV, RAV and ARF families. **b** Heatmap showing the expression profiles of B3 genes in different tissues, including four from sweet orange (leaf, fruit, callus and flower) and four from pummelo (leaf, fruit, ovule and seed). Color gradient from red-to-green indicates expression values change from high to low. **c** Structure of B3 genes with exon(s) in green, UTR regions in blue, and solid lines between the colored boxes indicating introns. The number indicates the phases of the corresponding introns. **d** Structures of B3 proteins with the B3 DNA binding domains represented by orange boxes, the AP2 domain in red, AUX/IAA in green, Auxin response factor in blue and CW-type zine finger domains represented by purple boxes

**Fig. 4:**
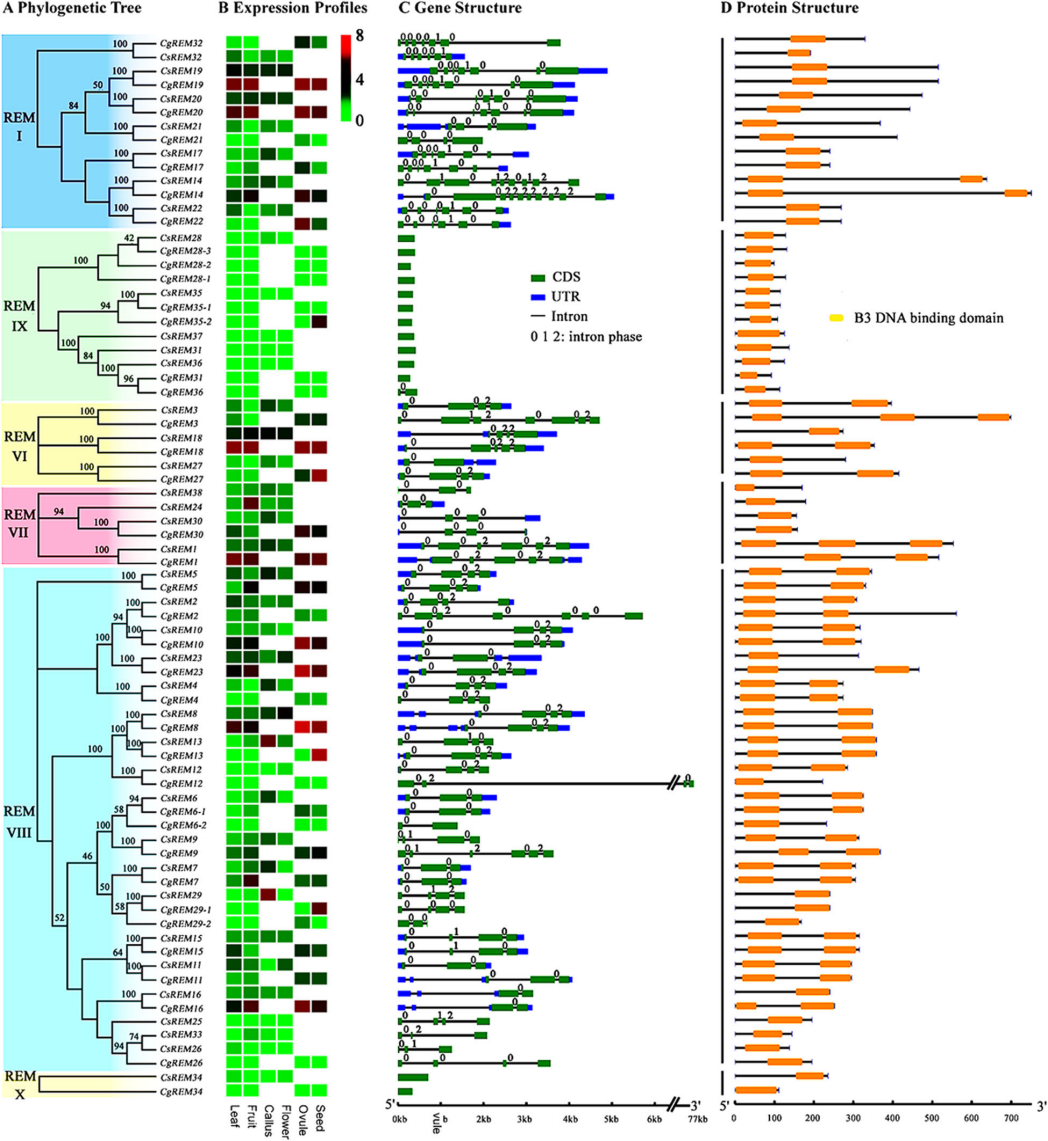
Phylogenetic relationships, expression profiles, gene structure and protein structure of citrus B3 genes from the REM family. **a** Neighbor-joining trees constructed for B3 genes from the REM family. **b** Heatmap showing the expression of B3 genes in different tissues, including four from sweet orange (leaf, fruit, callus and flower) and four from pummelo (leaf, fruit, ovule and seed). Color gradient from red-to-green indicates expression values change from high to low. **c** Structure of B3 genes with exon(s) in green, UTR regions in blue, and solid lines between the colored boxes indicating introns. The number indicates the phases of the corresponding introns. **d** Structure of B3 proteins with the B3 DNA binding domain(s) represented by orange boxes

The amino acids length of putative citrus B3 proteins varied widely, ranging from 93 to 1134 (Additional file 1). A few genes had short coding sequence lengths and showed very low expression levels in all samples studied (RPKM< 1 by RNA-Seq; RPKM: reads per kilobase per million mapped reads) (Figs. 3 and 4), indicating that they may be pseudogenes. The molecular weights and theoretical isoelectric points were also diverse (Additional file 1). The majority of B3 TFs contained only one B3 domain except for some REM family members (Figs. 3d and 4 d). A molecular modelling study was then undertaken using the known core structure of the B3 domain crystallized from AtFUS3 (Protein Data Bank code: 6j9b.2; Additional file 4) [45]. Our results showed that the crystal structure had a high degree of sequence identity (88.46%) to the experimentally determined template structure, suggesting that a reliable model was generated. The amino acid sequences alignments showed that the B3 domain sequences were highly conserved in LAV (overall GUIDANCE alignment score = 0.984), RAV (overall GUIDANCE alignment score = 0.906) and ARF families (overall GUIDANCE alignment score = 0.998) (Additional file 5), whereas the B3 domains of REM family exhibited a higher degree of divergence (overall GUIDANCE alignment score = 0.772) (Additional file 6). A total of 20, 38, and 24 highly conserved amino acid residues were identical among the B3 domains of all the LAV, RAV, and ARF family members, respectively (Additional file 5). For REM family members, only some conserved amino acid residues including one proline (position 31, P), two tryptophans (position 72 and 97, W), three glycines (position 70, 96 and 109, G) and three phenylalanines (position 34, 100 and 114, F) were observed in the B3 domains (Additional file 6), which indicated that the B3 domain might have been evolved independently in the REM family.

### Phylogenetic analyses of B3 genes

To explore the phylogenetic relationships of the B3 superfamily, an unrooted phylogenetic tree was constructed among the B3 genes of citrus (sweet orange and pummelo) and the model plant *Arabidopsis* (Additional file 7). In most subgroups, internal nodes were supported by confidence values of at least 70%, indicative of good consistency in the topology. The tree is in general agreement with *Arabidopsis* B3 superfamily trees published previously [1, 4], which further corroborates the reliability of the tree. In order to test the reliability of the tree topology, protein domain architecture (which was not used in the construction of the tree) were used to provide additional support for the proposed phylogeny. In addition to the B3 domain, other conserved motifs are highly clade specific (Fig. 3d). For example, the ARF and AUX/IAA motifs are specifically shared by ARF family. The distribution of the CW-type zinc finger motif supports the tree grouping of *CsLAV3*/*CgLAV3*, *CsLAV4*/*CgLAV4* and *CsLAV5*/*CgLAV5* together. Presence of the AP2 domain is also largely clade dependent in the RAV family. The fine structure of the trees is also supported by intron/exon structure data, with a few minor exceptions (Figs. 3c and 4c). For example, all the coding sequences of the ARF genes were disrupted by 2 to 15 introns, while the RAV family contained no more than one intron, except *CgRAV5*.

According to the classification criteria in *Arabidopsis*, we divided the members of the major four families into 14 major subgroups (Figs. 3a and 4 a). The LAV family could be subdivided into two subgroups, i.e. LEC2-ABI3 subgroup (I) and VAL subgroup (II). Four *CsLAVs* in sweet orange (*CsLAV1*, *CsLAV2*, *CsLAV6* and *CsLAV8*) and their counterparts in pummelo (*CgLAV1*, *CgLAV2*, *CgLAV6* and *CgLAV8*) were clustered with the *Arabidopsis* LEC2-ABI3 subgroup. The VAL subgroup of four citrus *LAV* genes (*CsLAV3/CgLAV3*, *CsLAV4/CgLAV4*, *CsLAV5/CgLAV5* and *CsLAV7/CgLAV7*), which had a conserved B3 domain and a CW-type zinc finger, were clustered with three *Arabidopsis* VAL proteins (Fig. 3 and Additional file 7).

The RAV family was grouped into two main subgroups based on their phylogenetic relationship. Subgroup I comprised three citrus RAV genes (*CsRAV1/CgRAV1*, *CsRAV2/CgRAV2* and *CsRAV4/CgRAV4*) that clustered with four *AtNGA* genes and three *AtRAV-like* genes from the same branch (Fig. 3a and Additional file 7). These genes commonly had the conserved B3 domain and contained no more than one intron (Fig. 3c and d). Subgroup II comprised of four *CsRAV* genes (*CsRAV3*, *CsRAV5*, *CsRAV6* and *CsRAV7*) and three *CgRAV* genes (*CgRAV3*, *CgRAV5* and *CgRAV6*), featuring a B3 domain with an upstream AP2 domain (Fig. 4d), and having no introns, except *CgRAV5* (Fig. 3c).

Citrus ARF genes were classified into four major subgroups. Subgroup I and II belonged to the same branch, and contained 6 members (*CsARF1/CgARF1*, *CsARF3/ CgARF3*, *CsARF5/CgARF5*, *CsARF11/CgARF11*, *CsARF17/CgARF17* and *CsARF18*) and 5 members (*CsARF2/CgARF2*, *CsARF7/CgARF7*, *CsARF8/CgARF8*, *CsARF15/CgARF15* and *CsARF16/CgARF16*), respectively (Fig. 3a and Additional file 7). Most of these genes were characterized as having a B3 DNA binding domain, ARF and AUX/IAA domains (Fig. 3d). Subgroup III (*CsARF4/CgARF4*, *CsARF6/CgARF6*, *CsARF10/CgARF10* and *CsARF19*) and Subgroup IV (*CsARF9/CgARF9*, *CsARF12/CgARF12-CsARF14/CgARF14*) only had the B3 and ARF domains*.* As most of the REMs in citrus possessed multiple B3 domains and shared low sequence similarity (Fig. 4d and Additional file 6), the phylogenetic analyses were performed within each subgroup of the REM family. The first step of the phylogenetic analysis was comparison of the AtREM sequences with CsREM/CgREM sequences according to the previous study [4] (Additional file 7). After this initial analysis, six common REM subgroups (REM I and REM VI to REM X) were identified between citrus and *Arabidopsis*, whereas REM V (*AtREM5*) was exclusively identified in *Arabidopsis*. The vast majority of subgroup I and subgroup II genes contained one B3 domain, and shared homology with the AtREM I and VII type genes, respectively (Fig. 4 and Additional file 7). Subgroup III and IV genes belonged to the AtREM IX and X types, respectively, which possessed only one B3 domain. Subgroup V (AtREM VI) and subgroup VI (AtREM VIII) genes contained several members, the majority of which had more than one B3 domain.

### Expression profiles of B3 genes in different tissues and during somatic embryogenesis

To understand the tissue expression profiles of the B3 genes in citrus, we compared their transcript abundance based on previously published RNA-seq data of different tissues including leaf, fruit, embryogenic callus, flower, ovule and seed from sweet orange and pummelo (Figs. 3b and 4 b). Many citrus B3 genes exhibited high transcript abundance level in all five tissues. However, the LEC2-ABI3 subgroup and two REM classes (REM IX type and REM X type) exhibited relatively lower expression levels compared with other *CsB3* genes. In addition, some of the B3 TFs exhibited tissue-specific expression. For example, *CsLAV1/2/6/7*, *CsARF9/19*, *CsREM3/4/6/7/9/13/14/17/27/28/29* showed the highest transcript abundance in the embryogenic callus (EC), whereas *CsREM24* was expressed predominantly in fruit. Some duplicated gene pairs also showed divergent expression profiles. For example, *CgARF13* showed a low expression level (RPKM = 2.76) in fruit; whereas its duplicated gene, *CgARF14*, was highly expressed (RPKM = 56.13) in fruit. These results suggest that duplicated genes may evolve to have diverse functions. Some clustered citrus B3 genes, which were identified as orthologous genes between sweet orange and pummelo species, showed different expression profiles. For example, *CgARF17* was mainly expressed in leaf (RPKM = 59.06) and ovule (RPKM = 57.40) of pummelo, whereas its orthologous gene (*CsARF17*) in sweet orange showed relatively low expression in all citrus tissues studied, with RPKM values ranging from 4.16 to 7.57.

**Fig. 5:**
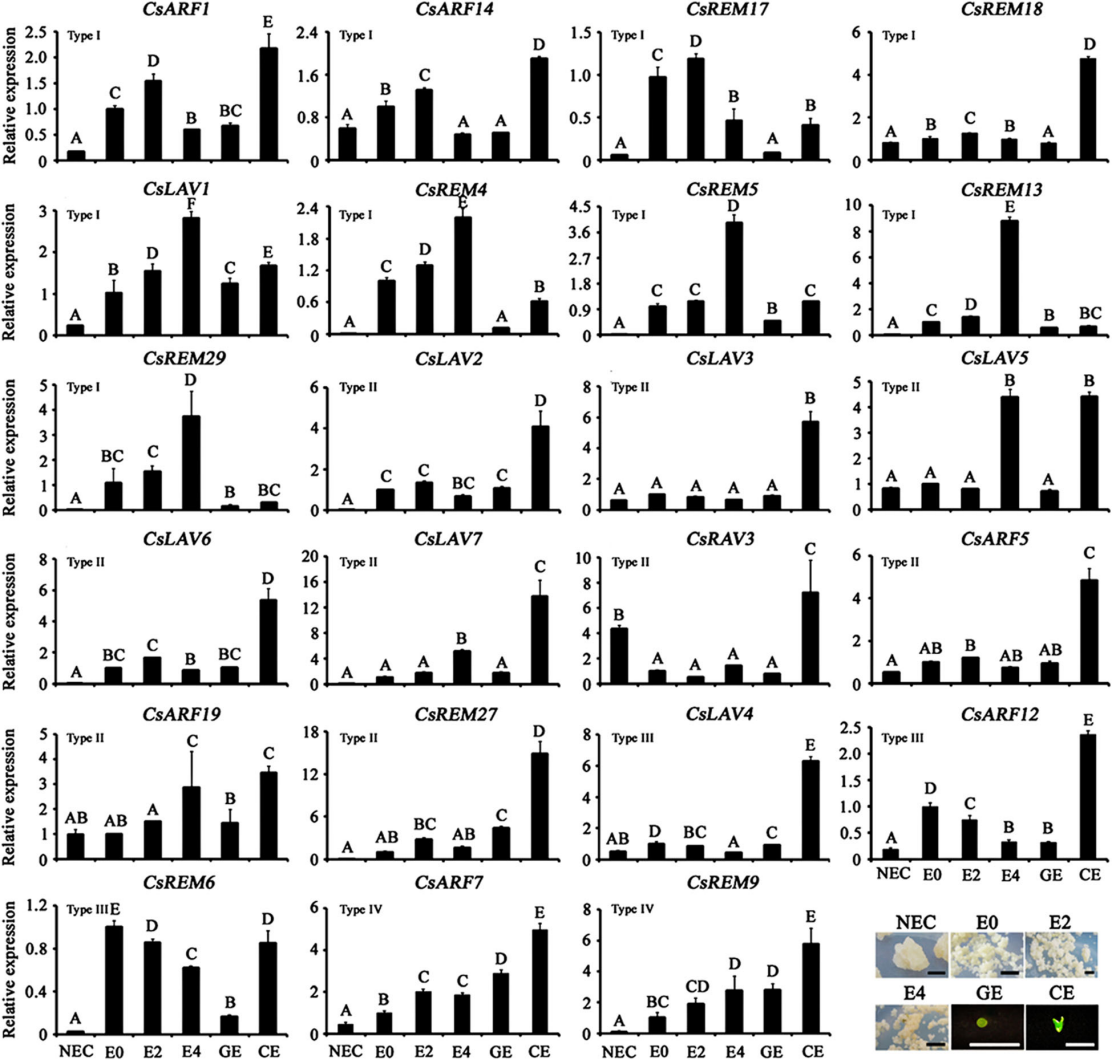
Expression profiles of 23 selected *CsB3* genes during somatic embryogenesis of ‘Valencia’ orange. Different capital letters above the bars indicate significant difference at *P* < 0.01. Non-embryogenic callus (NEC), embryogenic callus (EC) induced for somatic embryos for 0, 2, 4 weeks (E0, E2, E4), globular embryos (GE), cotyledon embryos (CE). Scale bar = 5 mm

To explore the possible involvement of *CsB3* genes during citrus SE, the expression profile of *23 CsB3* genes was investigated by qRT-PCR in the six SE stages of ‘Valencia’ orange, a citrus variety with strong SE capability. These genes were selected based on their relatively high transcript abundance (RPKM values > 10) in EC, or specific accumulation in EC with lower expression level (1 < RPKM values < 10) according to the RNA-seq data. Based on their expression profiles, these genes could be classified into four types (Fig. 5). The expression of Type I genes was up-regulated during differentiation and showed a highest peak value at the E2 stage (embryogenic callus induced for somatic embryos for 2 weeks; *CsARF1*, *CsARF14*, *CsREM17* and *CsREM18*) or E4 stage (embryogenic callus induced for somatic embryos for 4 week; *CsLAV1*, *CsREM4*, *CsREM5*, *CsREM13* and *CsREM29*), and then down-regulated at the early embryo morphogenesis stage (GE, globular embryos), whereas they showed another high peak at the late embryo morphogenesis stage (CE, cotyledon embryos). Type II genes comprise five *CsLAVs* (*CsLAV2*, *CsLAV3*, *CsLAV5*, *CsLAV6* and *CsLAV7*), one *CsRAV* (*CsRAV3*), two *CsARFs* (*CsARF5* and *CsARF19*) and one *CsREM* (*CsREM27*), and were specifically expressed highly at the CE stage, some of which also showed high transcript abundance in one other stage. For Type III genes (*CsLAV4*, *CsARF12* and *CsREM6*), the mRNA abundance was down-regulated during differentiation stages (E0-E4, embryogenic callus induced for somatic embryos for 0–4 weeks), but was higher at the subsequent stages of embryo morphogenesis (GE or CE). Genes in Type IV (*CsARF7* and *CsREM9*) increased progressively throughout the whole SE process.

**Fig. 6:**
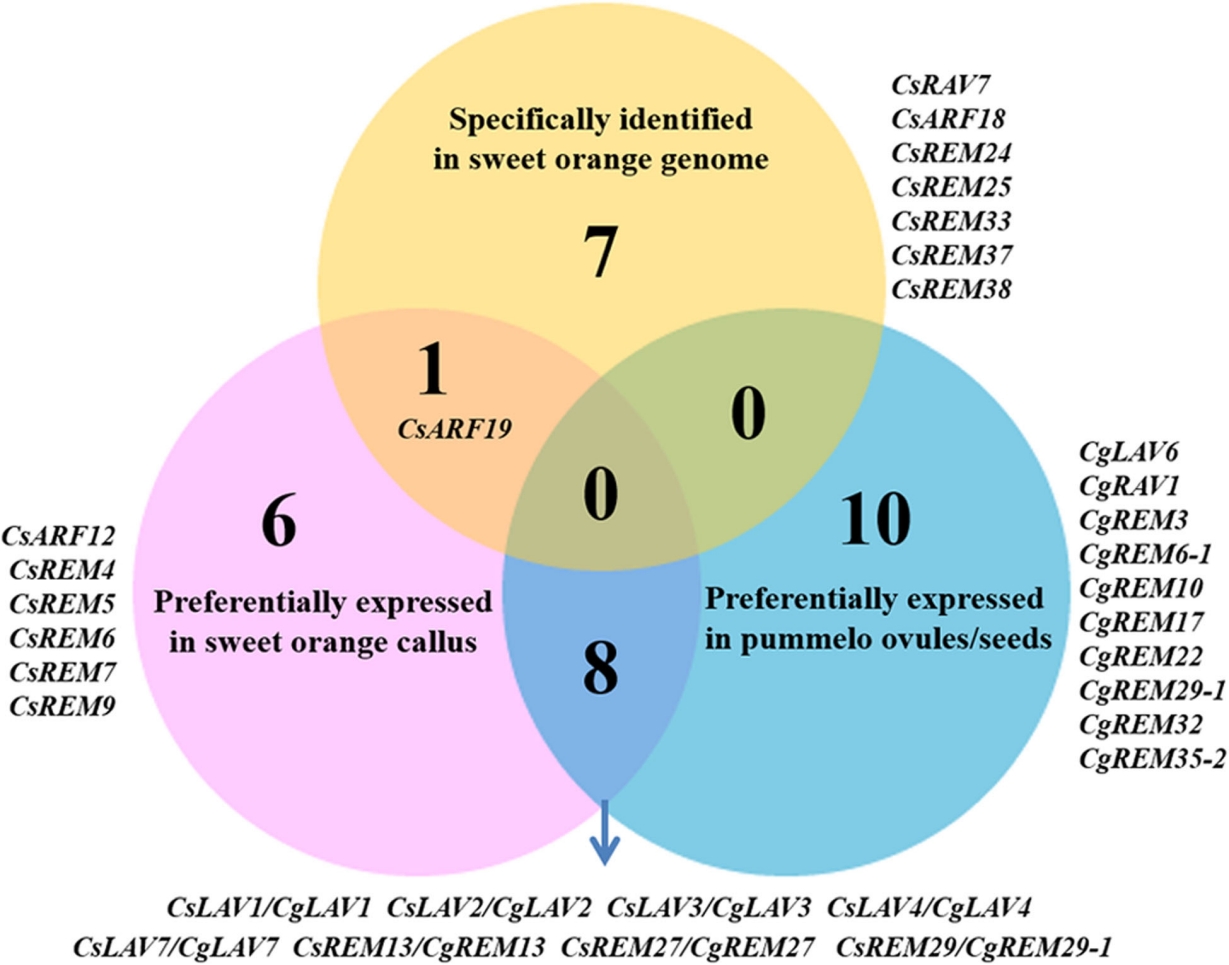
Overlap of citrus B3 genes in three datasets. The Venn diagram shows the overlap among the three B3 gene lists, i.e. genes specifically identified in the genome of sweet orange, genes preferentially expressed in the callus of sweet orange, and genes preferentially expressed in the ovule and/or seeds of pummelo, respectively. The genes in each group are listed

A total of 15 *CsB3* genes which were preferentially expressed in EC were retrieved from the RNA-seq data, including five *CsLAVs* (*CsLAV1* to *CsLAV4* and *CsLAV7*), two *CsARFs* (*CsARF12* and *CsARF19*) and eight *CsREMs* (*CsREM4* to *CsREM7*, *CsREM9*, *CsREM13*, *CsREM27*, *CsREM29*) (Fig. 3b, Figs. 4 b and 6). Among their orthologous genes, eight (five *CgLAVs*, *CgREM13*, *CgREM27* and *CgREM29–1*) were preferentially expressed in the ovules and/or seeds of pummelo (Fig. 6), suggesting that these genes may be associated with embryogenesis in vivo and in vitro. Meanwhile, eight B3 genes were identified in the genome of sweet orange, but not in that of pummelo, including *CsRAV7*, *CsARF18*, *CsARF19*, *CsREM24*, *CsREM25*, *CsREM33*, *CsREM37* and *CsREM38* (Fig. 6). Among them, *CsARF19* (Cs7g02210) showed markedly higher expression levels (≥6-fold) in EC compared with the other tissues (Fig. 3b), indicating its potential association with callus initiation, because empirically, EC can only be induced from the seeds of the polyembryonic citrus genotypes. With the availability of the citrus genome sequences [46–50], two orthologs of *CsARF19*, MSYJ162170.1 (amino acids sequence identity of 99.36%) and Ciclev10030751m (amino acids sequence identity of 99.87%), were identified in Mangshan mandarin (*C. reticulata*, a wild mandarin) and Clementine mandarin (*C. clementina*, which is believed to be a chance hybrid of mandarin and sweet orange) [48, 50, 51], respectively, but not in *Atalantia* (*Atalantia buxifolia*, a primitive citrus), Ichang papeda (*C. ichangensis*, a wild citrus) and three genera related to citrus, viz. Hongkong kumquat (*Fortunella hindsii*), trifoliate orange (*Poncitrus trifoliata*) and citron (*C. medica*).

## Discussion

The B3 superfamily is one of the largest and most diverse gene families in plants [1]. The evolution of the B3 superfamily has a long history, which can be traced back to the single-celled green algaes *Chlamydomonas reinhardtii* and *Ulva linza*, which possess a single B3 gene, strongly suggesting that the B3 domain arose before the development of multicellularity in the plant lineage [1]. In this study, we performed a comprehensive search for B3 superfamily genes throughout citrus genomes. A total of 72 *CsB3* and 69 *CgB3* genes were identified, accounting for 0.24% (29,445 predicted genes in sweet orange) and 0.23% (30,123 predicted genes in pummelo) of all predicted protein-coding genes [46, 47], which was lower compared to the result in *Arabidopsis thaliana* (110 *AtB3* genes out of the 25,498 predicted genes, accounting for 0.43%) [52], but higher than that in *Oryza sativa* (87 *OsB3* genes out of the 53,398 predicted gene, accounting for 0.16%) [53] (Additional file 8). These results showed that the B3 TFs unequally expanded with increasing genome size, since the sizes of the sweet orange and pummelo genomes (367 Mb and 380.76 Mb, respectively [46, 47]) are about triple of that of *Arabidopsis* (125 Mb) [52]. However, there were more identified *AtB3* genes in *Arabidopsis* than in citrus (Additional files 1 and 8), which probably was because of the lack of recent whole-genome duplication (WGD) event in citrus, whereas *Arabidopsis* has experienced two additional rounds of recent WGD events [46].

### Possible roles of *CsB3* genes during SE of citrus

Phylogenetic analysis showed that most of the B3 superfamily clades contained both citrus and *Arabidopsis* proteins (Additional file 7), suggesting that possible functions might be conserved between species. Together with expression pattern analysis of *CsB3* genes during SE, it may be possible to identify key regulators of this important process in citrus. Genes with a Type I expression pattern were preferentially expressed during initiation of citrus SE (E0-E4) (Fig. 5). E0-E4 stages are critical stages of differentiation when yellow-green proembryos are generated from the white-yellow EC. It has been demonstrated that *CsFUS3* gene (Cs2g14320), a type I *LAV* family member (*CsLAV1*), can enhance SE competence of the citrus EC partially by regulating SE-related TFs and hormone pathways, especially ABA and GA pathways [44]. In *Arabidopsis*, an ortholog (i.e., *AtARF5*) of another Type I gene *CsARF1* (Cs3g25860) is known to promote de novo shoot formation from *Arabidopsis* callus by pathways involving the downstream functions of *STM* and *CRF2* [54]. Thus, the elevated expression of the Type I B3 genes during SE suggested their possible involvement in citrus SE.

After the formation of GE, the embryoids develop into CE, each with two well-developed cotyledons, which denotes the end of embryo morphogenesis. The CEs undergo phase transition to generate a plantlet through a germination-like process. The majority, but not all, of the Type II and Type III *CsB3* genes showed progressively decreasing expression or maintained low levels of expression at EC differentiation stages (E0-E4), but had much higher expression in CE (Fig. 5). The well-studied B3 member *AtABI3*, which is the putative ortholog of the Type II gene *CsLAV2* (Cs5g34660) in *Arabidopsis*, has been demonstrated to regulate abscisic acid-responsive gene*s* in phase transition from late embryo development to germination [8, 55], implying that *CsLAV2* may also be functional in late embryogenesis.

The *AtLEC2* gene is the best recognized regulator of plant SE [9, 56]. However, in citrus SE, *CsLAV6* (Cs2g05780) (the Type II ortholog of *AtLEC2*) showed constitutively low expression during early stages of SE (E0-GE), and accumulated specifically in CE (Fig. 5). Previous studies have shown that *CsLAV6* was not present in suppression subtractive hybridization (SSH) libraries of citrus SE tissues [41], and expression of *CsLAV6* is not increased in *CsFUS3* overexpressed EC lines in which the SE competence was enhanced [44]. Thus, *CsLAV6* may not promote SE initiation like its ortholog *AtLEC2*, but have a function in late SE.

Three Type II B3 genes, namely *CsLAV3* (Cs6g10020), *CsLAV5* (Cs2g06770) and *CsLAV7* (Cs1g06390), clustered together and shared a high level of sequence similarity with three repressors of embryonic pathways in *Arabidopsis* (i.e., *AtVAL1*, *AtVAL2* and *AtVAL3*) (Additional file 7) [11]. AtVAL1–3 proteins are required for repression of LAFL TFs (i.e., *AtLEC1*, *AtABI3*, *AtFUS3* and *AtLEC2*) during germination, which is necessary for the transition from seed to seedling development [11, 57, 58]. *CsLAV3/5/7* were expressed specifically at late embryogenesis stages of citrus, i.e. CE and (or) E4 stages, suggesting their possible involvement in repression of the SE pathway, for transition to vegetative development.

Another Type II B3 gene *CsARF5* (Cs2g09440), the ortholog of *AtARF6*, showed constitutive expression levels in most of the tissues studied, but expression also peaked in CE (Fig. 5 and Additional file 7). As the cleavage target of miR167, *AtARF6* was reported to be required for SE formation, and the *arf6* mutant is severely inhibited for SE production in *Arabidopsis* [33]. In citrus, *CsARF5* has also been identified as the target of miR167 in leaf and fruit by degradome sequencing [59]. MiR167 showed low or undetectable expression levels in both EC and NEC (non-embryogenic callus), but accumulated in GE, and reached its peak in subsequently formed CE [40], with a similar expression pattern to *CsARF5* (Fig. 5). The accumulation of *CsARF5* in CE may suggest its involvement in late embryogenesis, whereas the non-antagonistic expression patterns between miR167 and *CsARF5* may result from fine-tuning of miRNA and/or post-transcriptional regulation.

During the SE process, the Type III B3 gene *CsARF12* was down-regulated progressively during the differentiation process (E0-E4), but up-regulated in CE (Fig. 5). It has been reported that *AtARF16*, an ortholog of *CsARF12* in *Arabidopsis*, regulates the expression of *AtABI3* in enhancing seed dormancy and ABA-mediated inhibition of seed germination [60]. The elevation of *CsABI3* (*CsLAV2*) and *CsARF12* expression levels at the CE stage might be involved in inhibition of germination at the late embryogenesis stages.

The expression level of two B3 genes (*CsARF7* and *CsREM9*), which were classified as Type IV, increased progressively during the citrus SE process (Fig. 5). *AtARF1*, which is homologous to *CsARF7* (Cs3g01570), binds to auxin response elements and confers auxin responsiveness in development [61]. In citrus, endogenous IAA levels likewise gradually increase during SE and reach a peak in CE [44]. However, the level of endogenous IAA was relatively lower in EC with greater potential for SE, suggesting that auxin may not be a key factor for determination of SE competence [44]. In addition to auxin, a high ratio of ABA to GA was shown to contribute to citrus SE. The accumulation of auxin has previously been reported to modulate the levels of ABA and GA in regions of future organogenesis [10]. Thus, the association of *CsARF7* and the plant hormone auxin, ABA and GA in citrus SE remains to be elucidated.

The *CsREM9* gene belongs to the poorly characterized REM family. Expression and genetic analyses showed that one REM gene (*AtVDD*) is required for cell differentiation in the female gametophyte and is highly expressed during early stages of seed formation [39]. SE shares morphological, cytological, and molecular similarities with zygotic embryogenesis (ZE) [44, 62]. The increased expression of *CsREM9* during the early stages of SE suggests that *CsREM9* may be functional in early embryogenesis as well (Fig. 5).

### *CsARF19* might be involved in citrus EC initiation

In citrus, EC can be induced in vitro from the undeveloped ovules/ aborted seeds of the polyembryonic genotypes, but not from the monoembryonic genotypes, which suggests that the regenerative EC might be derived from pluripotent nucellar embryo initiation (NEI) cells localized in the apomictic nucellus tissues [63]. In this study, we identified a candidate B3 TF possibly involved in EC initiation (Fig. 6). *ARF19* was identified in mandarin and sweet orange, but not in pummelo or related genera of citrus. In addition, *CsARF19* was expressed at a relatively high level in EC compared to other tissues in sweet orange (Fig. 3b). In our previous study, *ARF19* was moderately expressed (RPKM values ranged from 15.69 to 34.50) in the ovules of two mandarin cultivars (the monoembryonic ‘Nour’ Clementine and the polyembryonic ‘Huagan No.2’ Ponkan) during nucellar embryo initiation, but was expressed at slightly higher levels in the polyembryonic cultivar [64]. However, *ARF19* was not expressed (RPKM = 0) in ovules of the monoembryonic ‘Huanong red’ pummelo, but accumulated in ovules of the polyembryonic ‘Cocktail’ grapefruit, with RPKM values of 1.34 and 3.58 prior to and during nucellar embryo initiation [64]. Based on the fact that sweet orange and grapefruit were derived from hybridizations between mandarin and pummelo [46, 51, 64], we suggest that *CsARF19* originated in mandarin, and was introgressed into the hybrid pool. SE initiation is believed to require an induction signal that causes somatic cells to change identity [65]. Previous studies have shown that two *ARF* genes (*AtARF7* and *AtARF19*) directly or indirectly target four auxin-responsive LBD (LATERAL ORGAN BOUNDARIES DOMAIN) genes to regulate callus formation in *Arabidopsis* regeneration [66]. Callus-induction medium containing a high concentration of 2,4-dicholorophenoxy acetic acid (2,4-D) has also been shown to promote callus induction in citrus [67, 68]. However, the link between auxin signaling and citrus callus initiation has not yet been established. Our analysis implies that *CsARF19* is derived from mandarin, a basic species of citrus, and may be involved in callus initiation process from the nucellus tissues of polyembryonic citrus cultivars.

Overall, the present study indicates that some given members of the B3 superfamily may be involved in citrus SE, especially for late SE stages. One B3 gene, *CsARF19*, was indicated to be associated with nucellar-derived callus initiation of polyembryonic citrus cultivars. Although phylogenetic and expression analysis provided clues to the roles of the citrus B3 superfamily in SE, further molecular and biochemical studies are required to investigate whether multiple members of B3 superfamily form a coordinated regulation network to control SE, and whether the expression of some key B3 genes could establish a cellular environment favorable to callus initiation or SE competence enhancement.

## Methods

### Plant materials

Non-embryogenic callus (NEC) and embryogenic callus (EC) of ‘Valencia’ sweet orange were induced, cultured and preserved as described previously [69]. The plant material was identified by Dr. Zheng Liu, an associate researcher of Fruit and Tea Research Institute, Hubei Academy of Agricultural Sciences. The voucher specimens were deposited at Key Laboratory of Horticultural Plant Biology (Ministry of Education), Huazhong Agricultural University. In brief, NEC was recently induced from epicotyl segments, whereas EC was induced from the aborted seeds and preserved in tissue culture for years. The seeds were harvested from ‘Valencia’ sweet orange trees in the citrus germplasm respository at Huazhong Agricultural University. EC was transferred to glycerol medium to induce SE. Samples were collected from NEC, EC, E2/4 (EC induced from somatic embryos for 2 or 4 weeks), GE and CE. All samples were immediately frozen in liquid nitrogen and stored at − 80 °C for further analysis.

### Genome-wide identification of B3 superfamily genes

A HMM (Hidden Markov Model) profile of the B3 DNA binding domain (PF02362) was downloaded from the Pfam database (http://pfam.xfam.org/family/PF02362), and exploited for the comprehensive identification of sweet orange and pummelo B3 superfamily genes from the *Citrus sinensis* Annotation Project (http://citrus.hzau.edu.cn/orange/download/index.php) using HMMER program (version 3.1b2) with a threshold of e-value < 0.01 [46, 47]. Using the same criterion, B3 family sequences were obtained from *Arabidopsis thaliana* (ftp://ftp.ensemblgenomes.org/pub/plants/release-38/fasta/arabidopsis_thaliana/) and *Oryza sativa* databases (ftp://ftp.ensemblgenomes.org/pub/plants/release-38/fasta/oryza_sativa/). The conserved domains of all putative candidates were confirmed using InterProScan software package (version 5.25–64.0). Finally, a self-blast of protein sequences was performed to remove redundancy. Alternative splice variants were not considered. Any two protein sequences which showed a perfect match were deemed to be redundant gene pairs and the shorter sequence was removed from the potential B3 genes list. Seven draft genomes of citrus species (http://citrus.hzau.edu.cn/orange/download/index.php), including Mangshan mandarin, Clementine mandarin, *Atalantia*, Ichang papeda, kumquat, trifoliate orange (unpublished data) and citron [47–50], were used to search for orthologs of *CsARF19*.

### Analysis of chromosomal locations, synteny relationships, protein properties, gene structure and conserved motifs

The physical locations of citrus B3 genes were obtained from the database of sweet orange and pummelo genomes (http://citrus.hzau.edu.cn/orange/download/index.php). MapChart software (https://www.wur.nl/en/show/Mapchart.htm) was applied to visualize the distribution of the B3 genes on the citrus chromosomes. To detect gene duplication events, the Multiple Collinearity Scan toolkit (MCScan X) was applied [70]. Dual Synteny Plotter software (https://github.com/CJ-Chen/TBtools) was adopted to exhibit the synteny relationship of the orthologous B3 genes between citrus and *Arabidopsis* as well as between citrus and rice. The Ks and Ka were calculated using KaKs_Calculator 2.0 [71].

The theoretical isoelectric points and molecular weights of the citrus B3 proteins were predicted by the compute pI/Mw tool in the ExPASY server (https://web.expasy.org/compute_pi/). The Gene Structure Display Server (GSDS, http://gsds.cbi.pku.edu.cn/) program was exploited to illustrate exon/intron organization according to cDNA and genomic DNA sequences. The InterProScan program (http://www.ebi.ac.uk/interpro/) was used to characterize the domains and motifs of the citrus B3 superfamily. The predicted three-dimensional structure of the citrus B3 domain was generated using the Swiss-Model server by homology modeling [72]. The B3 domain of *CsLAV1* (*CsFUS3*; gene id: Cs2g14320) was chosen as a template, based on the known structure of AtFUS3 (Protein Data Bank code: 6j9b.2) [45].

### Multiple sequence alignments and phylogenetic analysis

Multiple sequence alignments (MSA) of the B3 domain sequences of citrus B3 proteins were performed using the MAFFT algorithm (version 7) [73]. The robustness of the MSA was assessed by the GUIDANCE2 server (http://guidance.tau.ac.il/ver2/) using 100 bootstrap replicates [74]. The alignment was also presented along with the corresponding secondary structure elements.

To investigate the phylogenetic relationship between citrus and *Arabidopsis*, neighbor-joining (NJ) trees were constructed by MEGA7 software based on the full-length of the B3 protein sequences [75]. A bootstrap analysis was performed to estimate the reliability of the tree topology, with 1000 replications. The numbers generated for each clade represent the bootstrap support values expressed as percentages. The same method was adopted to construct the NJ phylogenetic trees for the four families of citrus B3 superfamily.

### Expression analysis of *CsB3s*

To investigate the expression patterns of all B3 genes in different citrus tissues, the normalized RPKM values of citrus B3 genes were extracted from the previously published RNA-Seq data of leaf, fruit, callus and flower of sweet orange [46] and that of leaf, fruit, ovule and seed of pummelo [47]. The results were visualized by heat map with transformed log_2_ (RPKM+ 1) values using the ‘pheatmap’ R package (https://cran.r-project.org/web/packages/pheatmap/index.html).

Genes that were highly expressed (RPKM values > 10) in EC, or specifically accumulated in EC with lower expression level (1 < RPKM values < 10) were selected for further analysis using qRT-PCR. Total RNA was extracted using the Trizol reagent from EC and somatic embryos of ‘Valencia’ sweet orange [69], followed by RNA integrity examination on 1.0% agarose gels stained with ethidium bromide. First strand cDNA was synthesized using the RevertAid™ First Strand cDNA Synthesis Kit (Fermentas, USA). qRT-PCR was performed as described previously [69]. qRT-PCR primer pairs were designed by Primer Premier 5.0 software (Additional file 9). The specificity of the primers was further confirmed with a melting curve analysis after amplification of each tested genes. Each PCR pattern was verified using four biological replicates. Two reference genes, i.e. *CiteIF-1A* and *CitUBL5*, shown to be stably expressed during citrus SE [69], were used as internal controls to normalize the qRT-PCR data. Mixtures without template were employed as the negative control. Data was processed using the Ct method (2^-△△*C*T^) for relative quantification. Statistical analyses were performed using the IBM SPSS Statistics 19 software as described previously [44].

## Supplementary information

**Additional file 1 **B3 genes identified in the genomes of sweet orange (*Citrus sinensis*) and pummelo (*C. grandis*).

**Additional file 2 **Ka/Ks calculation of the duplicated B3 gene pairs in sweet orange (*Citrus sinensis*) and pummelo (*C. grandis*).

**Additional file 3 **One-to-one orthologous relationships between sweet orange/pummelo and *Arabidopsis* as well as those between sweet orange/pummelo and rice.

**Additional file 4.** Three-dimensional models of the B3 domain from citrus. The Ribbon diagram of the CsLAV1 B3 domain was built using the SWISS-MODEL server. The B3 domain consists of seven β-strands (β1-β7) that form an open β-barrel. Two α-helices (α1 and α2) project above and below the β-barrel.

**Additional file 5.** Multiple-sequence alignments of the B3 domain from LAV, RAV and ARF families of citrus. Red circles indicate identical amino acid residues. Color-coded GUIDANCE2 scores are presented for the citrus B3 domain sequences. Confidently aligned residues are colored in shades of magenta, whereas uncertain residues are colored in shades of blue. GUIDANCE2 scores which represent the degree of confidently aligned residues (1 corresponds to 100% certainty) are plotted below the alignment.

**Additional file 6.** Multiple-sequence alignments of the B3 domain from the REM family of citrus. Red circles indicate conserved amino acid residues. Color-coded GUIDANCE2 scores are presented for the citrus B3 domain sequences. Confidently aligned residues are colored in shades of magenta, whereas uncertain residues are colored in shades of blue. GUIDANCE2 scores which represent the degree of confidently aligned residues (1 corresponds to 100% certainty) are plotted below the alignment.

**Additional file 7 **Phylogenetic tree of the B3 proteins from citrus and *Arabidopsis* based on the neighbor-joining method using MEGA7 software. The reliability of the predicted tree was tested by bootstrapping with 1000 replicates. The percentage of neighbor-joining bootstrap replications (> 40%) is shown above each node.

**Additional file 8 **Lists of B3 genes identified in *Arabidopsis* and rice.

**Additional file 9.** List of qRT-PCR primers used in this study.

## Data Availability

The sequences information analyzed during the current study are available in the *Citrus sinensis* Annotation Project (http://citrus.hzau.edu.cn/orange/download/index.php), *Arabidopsis thaliana* databases (ftp://ftp.ensemblgenomes.org/pub/plants/release-38/fasta/arabidopsis_thaliana/) and *Oryza sativa* databases (ftp://ftp.ensemblgenomes.org/pub/plants/release-38/fasta/oryza_sativa/). The public RNA-seq data was from the *Citrus sinensis* Annotation Project and NCBI’s Sequence Read Archive (SRA) database (the accession number PRJNA339650). All data analyzed during this study are included in this published article and its supplementary information files.

## Abbreviations

SE: Somatic embryogenesis
EC: Embryogenic callus;
TFs: Transcription factors
RPKM: Reads per kilobase per million mapped reads
Ka/Ks: Non-synonymous/synonymous substitution ratio
NEC: Non-embryogenic callus
E0/2/4: EC induced for somatic embryos for 0, 2, 4 weeks
GE: Globular embryos
CE: Cotyledon embryos
WGD: Whole-genome duplication
NJ: Neighbor-joining
MSA: Multiple sequence alignments.
