# Supplementary figures and images for: Genome-wide analysis of the citrus B3 superfamily and their association with somatic embryogenesis
Source: BMC Genomics. 2020 Apr 16;21:305. doi: 10.1186/s12864-020-6715-9 (PMC7161213; doi:10.1186/s12864-020-6715-9)

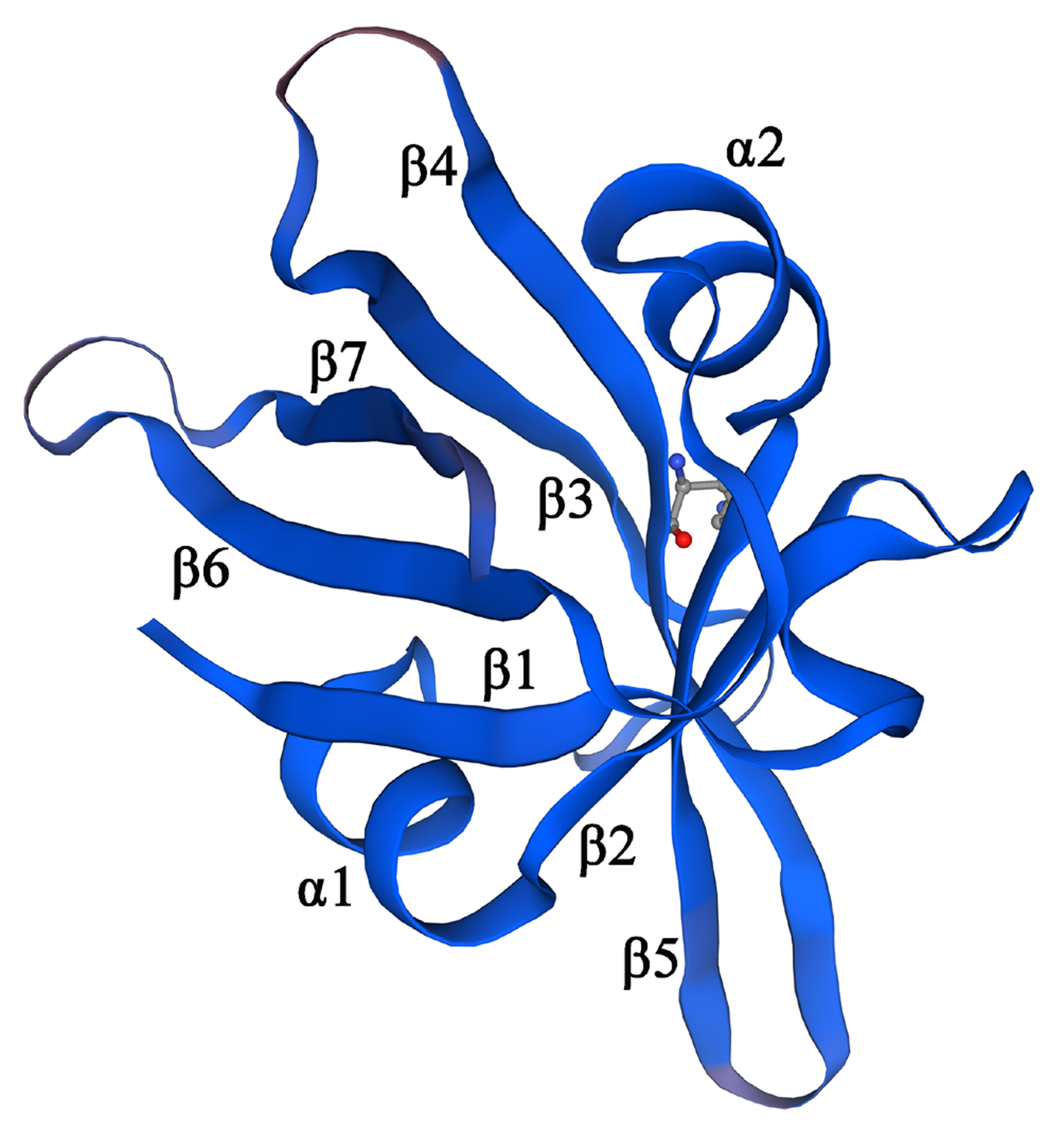

Supplement: Supplementary file 4 — Additional file 4. Three-dimensional models of the B3 domain from citrus. The Ribbon diagram of the CsLAV1 B3 domain was built using the SWISS-MODEL server. The B3 domain consists of seven β-strands (β1-β7) that form an open β-barrel. Two α-helices (α1 and α2) project above and below the β-barrel. [file 12864_2020_6715_MOESM4_ESM.tif]

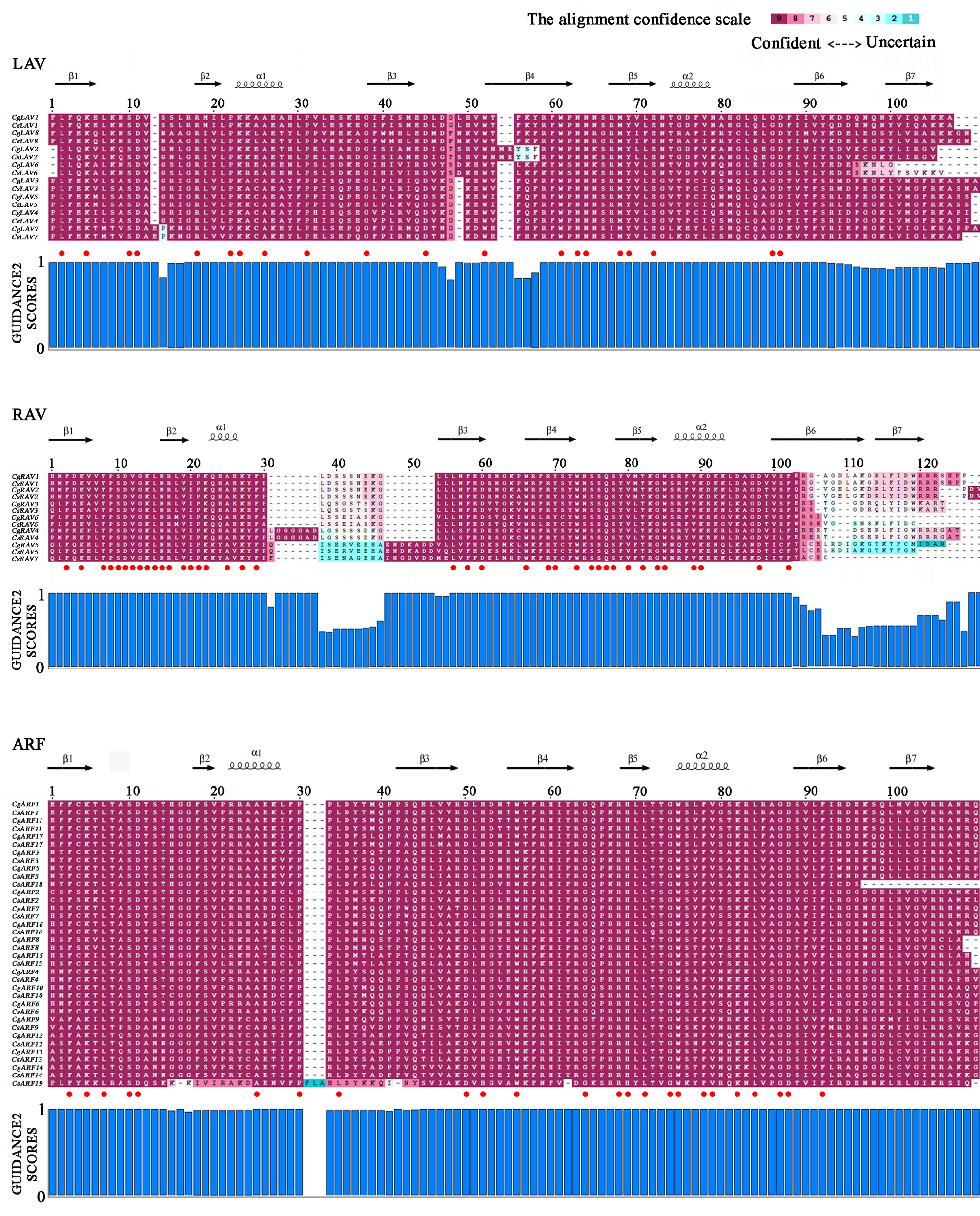

Supplement: Supplementary file 5 — Additional file 5. Multiple-sequence alignments of the B3 domain from LAV, RAV and ARF families of citrus. Red circles indicate identical amino acid residues. Color-coded GUIDANCE2 scores are presented for the citrus B3 domain sequences. Confidently aligned residues are colored in shades of magenta, whereas uncertain residues are colored in shades of blue. GUIDANCE2 scores which represent the degree of confidently aligned residues (1 corresponds to 100% certainty) are plotted below the alignment. [file 12864_2020_6715_MOESM5_ESM.tif]

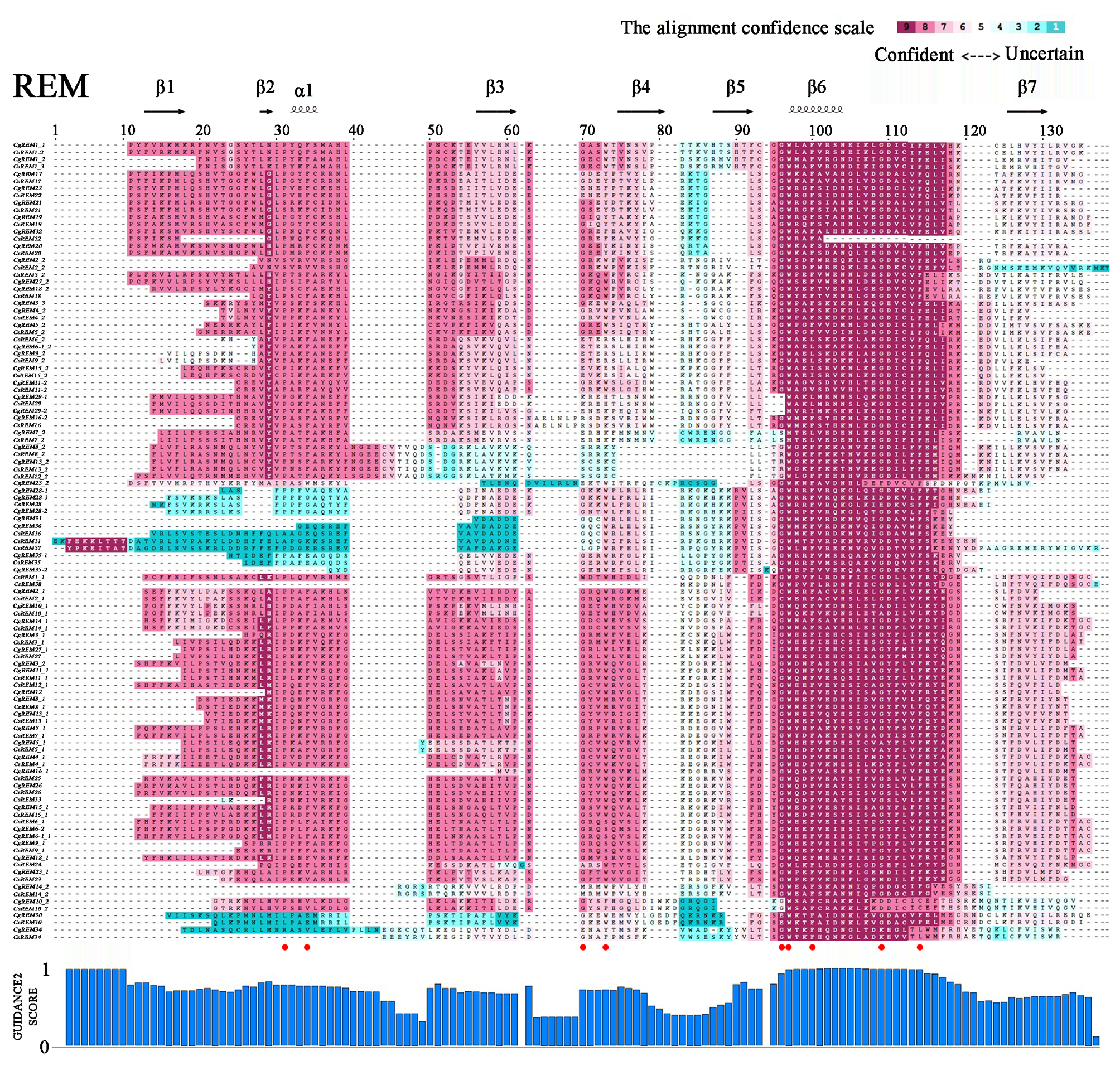

Supplement: Supplementary file 6 — Additional file 6. Multiple-sequence alignments of the B3 domain from the REM family of citrus. Red circles indicate conserved amino acid residues. Color-coded GUIDANCE2 scores are presented for the citrus B3 domain sequences. Confidently aligned residues are colored in shades of magenta, whereas uncertain residues are colored in shades of blue. GUIDANCE2 scores which represent the degree of confidently aligned residues (1 corresponds to 100% certainty) are plotted below the alignment. [file 12864_2020_6715_MOESM6_ESM.tif]

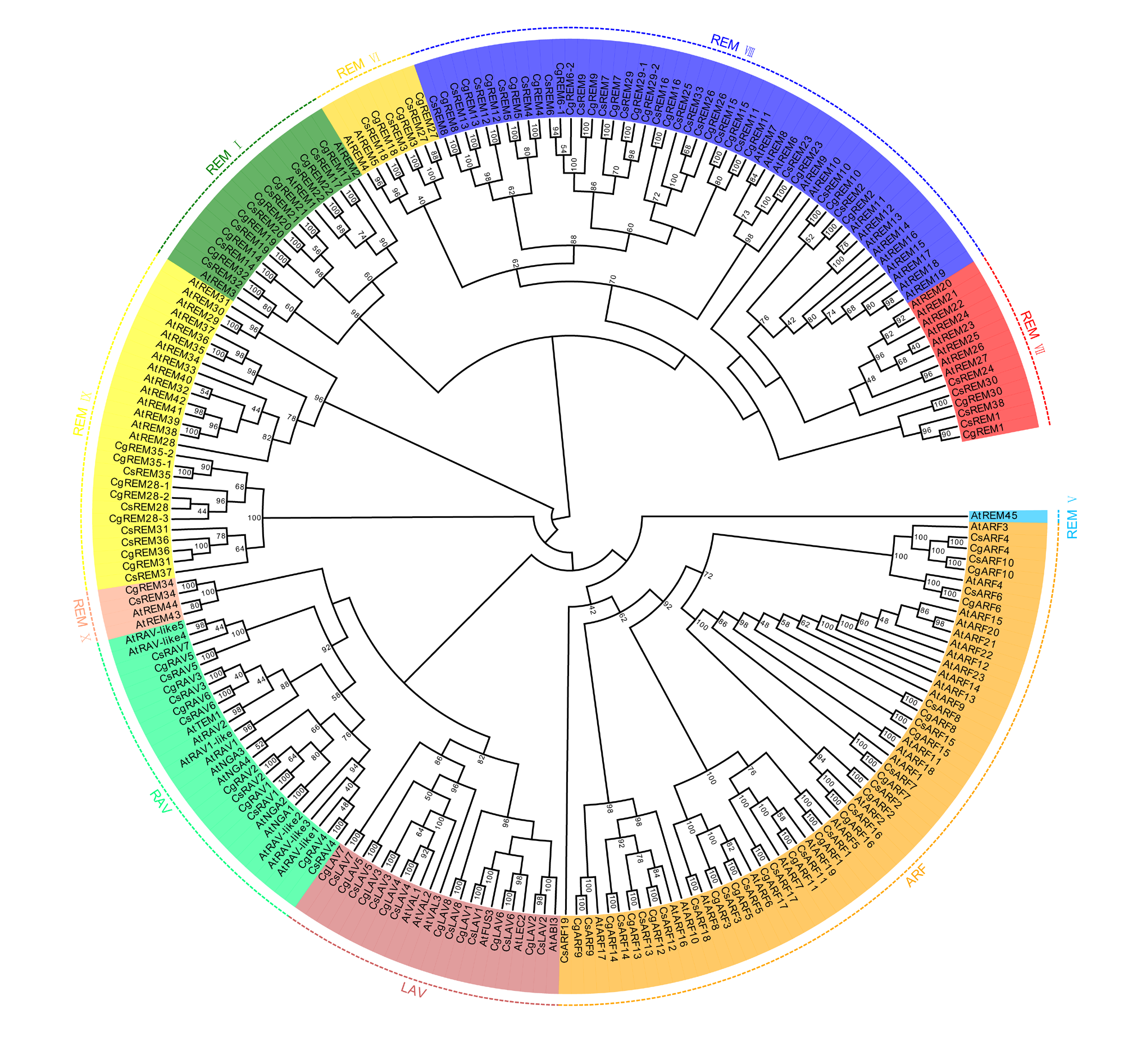

Supplement: Supplementary file 7 — Additional file 7 Phylogenetic tree of the B3 proteins from citrus and Arabidopsis based on the neighbor-joining method using MEGA7 software. The reliability of the predicted tree was tested by bootstrapping with 1000 replicates. The percentage of neighbor-joining bootstrap replications (> 40%) is shown above each node. [file 12864_2020_6715_MOESM7_ESM.tif]
