# Supplementary material for: Genome-wide analysis of the citrus B3 superfamily and their association with somatic embryogenesis
Source: BMC Genomics. 2020 Apr 16;21:305. doi: 10.1186/s12864-020-6715-9 (PMC7161213; doi:10.1186/s12864-020-6715-9)
Supplement: Supplementary file 9 — Additional file 9. List of qRT-PCR primers used in this study. [file 12864_2020_6715_MOESM9_ESM.doc]

**Additional file 9: List of qRT-PCR primers used in this study.**

| **Gene name** | **Primer sequences (5’-3’)** | |
| --- | --- | --- |
|  | **Forward** | **Reverse** |
| *CsLAV1* | CCGGCATGTCGTTTATTTATGAC | GAACCCCCCAAAAAATCCA |
| *CsLAV2* | AAGACTTTCAACGCACCC | GTCCGCATTCTGACCCT |
| *CsLAV3* | CTGGTTGGAGGAAGTGT | GAATCAAAGAAAGGGAAG |
| *CsLAV4* | GAGTTGGGTGTAGGAGC | ATAGGAGTTTGCGTGTC |
| *CsLAV5* | ACTTCTCCGTCCACCTC | TCAAACTCATACCCTTCA |
| *CsLAV6* | TAACCAGCAACTTAGCC | AAAGATTGACCACCAGA |
| *CsLAV7* | CTGGGACTCAGAAGGA | CAGGCAAACAAGCATC |
| *CsRAV3* | ATGGACGAAAGCACGAC | CGGAGGGAAAGTTATTGG |
| *CsARF1* | GCAAGCCTAGCAGATTC | AAGATGTGCCACCAGAG |
| *CsARF5* | CCTGCGACAAATCCTAT | GCTTCTCGGCAAGTTAA |
| *CsARF7* | AAAACTTCCAGGTAACGA | CACCCAGGTCCAACAAT |
| *CsARF12* | AAAAGTTGCGGCTTCCT | TGCCTGGCTCCCTGTAT |
| *CsARF14* | AGAGTGAAGCCTGAGATG | GCACGAGGATAATAAACA |
| *CsARF19* | TCCCTTCCAATAAACC | TCCAAACAATCCCAAA |
| *CsREM4* | GATCTGTATTAGTGGAGCAT | AAGCCGAAACAATCTG |
| *CsREM5* | ATTGTCGGCTTTGCTT | GTCTTTCTTCCGCTTC |
| *CsREM6* | TGGTGGACAGTGAGGA | TTTACGCTTCTTGCTT |
| *CsREM9* | TGGAGAAATGGGAATA | TCAATGATGTGGCAAT |
| *CsREM13* | TGGAGGTGAATGATGAA | TACTTTGGCGTGAACAG |
| *CsREM17* | AGCCCTCTATTGAACC | GACAAATCCCTCTTGG |
| *CsREM18* | TTTCAATCCTCCGTCAT | ATCTTTCGCTTTCTCCC |
| *CsREM27* | TTCAGTGGTGGAACAAA | AAGCCAATAGGAAACGA |
| *CsREM29* | GAGAATGAAACCCAATA | AGCAGCGTTAGAAAGT |
| *CiteIF-1A* | CCCCAAAGAATCAGAAACCAT | TGCCGAGCATACGAAGGAC |
| *CitUBL5* | CAAGGACTACGAGATTCACGAC | CCTGCCGCATCTTCCAAT |
